# The Root Extract of *Scutellaria baicalensis* Induces Apoptosis in EGFR TKI-Resistant Human Lung Cancer Cells by Inactivation of STAT3

**DOI:** 10.3390/ijms22105181

**Published:** 2021-05-13

**Authors:** Hyun-Ji Park, Shin-Hyung Park, Yung-Hyun Choi, Gyoo-Yong Chi

**Affiliations:** 1 Department of Pathology, College of Korean Medicine, Dong-eui University, Busan 47227, Korea; 14554@deu.ac.kr (H.-J.P.); cgyu@deu.ac.kr (G.-Y.C.); 2 Department of Biochemistry, College of Korean Medicine, Dong-eui University, Busan 47227, Korea; choiyh@deu.ac.kr

**Keywords:** *Scutellaria baicalensis*, lung cancer, EGFR TKI resistance, apoptosis, STAT3

## Abstract

Resistance to epidermal growth factor receptor tyrosine kinase inhibitors (EGFR TKIs) is a major obstacle in managing lung cancer. The root of *Scutellaria baicalensis* (SB) traditionally used for fever clearance and detoxification possesses various bioactivities including anticancer effects. The purpose of this study was to investigate whether SB exhibited anticancer activity in EGFR TKI-resistant lung cancer cells and to explore the underlying mechanism. We used four types of human lung cancer cell lines, including H1299 (*EGFR* wildtype; EGFR TKI-resistant), H1975 (acquired TKI-resistant), PC9/ER (acquired erlotinib-resistant), and PC9/GR (acquired gefitinib-resistant) cells. The ethanol extract of SB (ESB) decreased cell viability and suppressed colony formation in the four cell lines. ESB stimulated nuclear fragmentation and the cleavage of poly(ADP-ribose) polymerase (PARP) and caspase-3. Consistently, the proportion of sub-G1 phase cells and annexin V+ cells were significantly elevated by ESB, indicating that ESB induced apoptotic cell death in EGFR TKI-resistant cells. ESB dephosphorylated signal transducer and activator of transcription 3 (STAT3) and downregulated the target gene expression. The overexpression of constitutively active STAT3 reversed ESB-induced apoptosis, suggesting that ESB triggered apoptosis in EGFR TKI-resistant cells by inactivating STAT3. Taken together, we propose the potential use of SB as a novel therapeutic for lung cancer patients with EGFR TKI resistance.

## 1. Introduction

Lung cancer is a leading cause of cancer-related mortality worldwide, accounting for 25% of all cancer deaths [1]. Although the treatment for lung cancer has advanced significantly in recent decades, the 5-year survival rate of lung cancer is still less than 20% [1,2]. Late-stage diagnosis is related to the poor prognosis of patients with lung cancer. Almost 80% of lung cancer is diagnosed when local invasion or distal metastases has already occurred and the 5-year survival rate for patients with stage Ⅳ lung cancer is only 5% [1,2]. Platinum-based chemotherapy is the principal treatment for advanced non-small-cell lung cancer (NSCLC) [3]. However, chemotherapy drugs can cause a variety of side effects, and the response rate drops when drug resistance develops [4]. Therefore, it is fundamental to develop novel therapeutic strategies for the treatment of lung cancer.

*Epidermal growth factor receptor (EGFR)* mutations are found in approximately 38% of NSCLC patients. *EGFR* mutations are more frequent in females, non-smokers, and Asian patients [5]. Most *EGFR* mutations are either short deletions in exon 19 or an L858R substitution in exon 20 [6], and these *EGFR*-activating mutations subsequently stimulate EGFR downstream signaling pathways, which leads to the proliferation and invasion of cancer cells [7]. Notably, first-generation EGFR tyrosine kinase inhibitors (TKIs), including gefitinib and erlotinib, significantly reduced the tumor size and improved the overall survival of NSCLC patients with *EGFR* mutations [8,9,10,11]. Clinical guidelines also recommended that *EGFR* mutation-positive patients received first-line therapy with EGFR TKIs [12,13]. Despite the benefits of EGFR TKIs, acquired resistance develops within one year of treatment and weakens the efficacy of these drugs [14]. The most common mechanism of the resistance to EGFR TKIs is the *EGFR* T790M mutation, which accounts for about 60% of the cases with acquired resistance [15]. The activation of alternative pathways, such as MET amplification or HER2 upregulation, impairment of the pathway involved in the EGFR TKI-induced apoptosis, such as BIM regulation, and histologic transformation are also considered to confer EGFR TKI resistance [16]. Although next-generation EGFR TKIs, including afatinib, an irreversible EGFR inhibitor, and osimertinib, the third-generation TKI that targets the *EGFR* T790M mutation, have been developed to overcome EGFR TKI resistance, the clinical benefit is limited and additional resistance to these drugs has been reported [17,18]. Another strategy is combination therapy, which is to add a new agent to EGFR TKIs. However, combination therapy is unlikely to overcome the resistance derived from the *EGFR* T790M mutation and shows overlapping toxicities [16]. Therefore, the development of new therapeutic drugs for the treatment of lung cancer patients with EGFR TKI resistance is urgently needed.

The root of *Scutellaria baicalensis* (SB) is one of the fundamental herbs used in traditional Oriental medicine. According to traditional herbology, it plays a role in clearing heat and drying dampness. It has been applied in the treatment of hepatitis, hypertension, and acute infections of the respiratory or gastrointestinal tracts for thousands of years [19,20]. Recent studies have reported that SB possessed various pharmacological activities, such as neuroprotective, liver protective, anti-inflammatory, antibacterial, and antioxidant effects [19,20]. Furthermore, SB exhibited anticancer activities in a wide range of cancer cells [20]. The SB extracts not only suppressed cell cycle progression and migration, but also induced apoptosis in human lung cancer cells [21,22,23,24]. However, the anticancer effects of SB in EGFR TKI-resistant lung cancer cells, and the role of signal transducer and activator of transcription 3 (STAT3) in the SB-induced apoptosis have not been elucidated yet. In this study, we investigated whether SB exerted anticancer activities in EGFR TKI-resistant lung cancer cells and explored the underlying mechanism.

## 2. Results

### 2.1. Identification of Baicalin in the Ethanol Extract of SB by HPLC-MS Analysis

**Figure 1:**
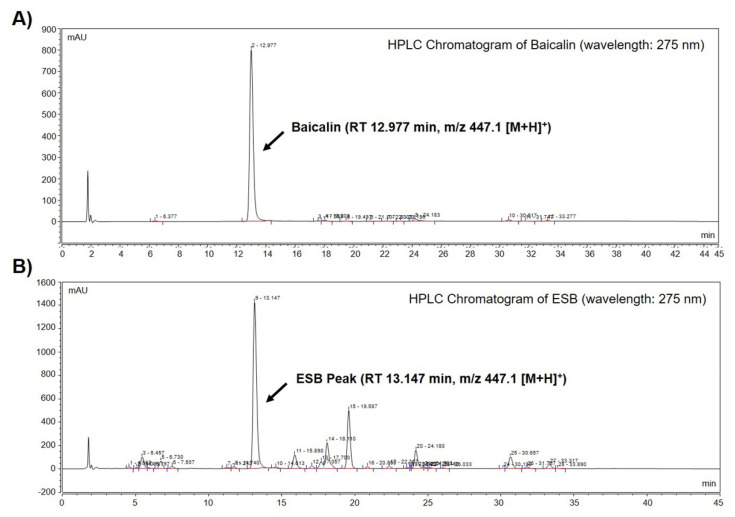
Identification of baicalin from ESB by HPLC-MS analysis. Total HPLC-chromatograms of baicalin (**A**) and ESB (**B**) were obtained at a UV wavelength of 275 nm. The molecular weight of the indicated peak in the chromatogram of baicalin (**A**) and ESB (**B**) was measured by HPLC-MS/MS analysis. ESB, ethanol extract of the root of *Scutellaria baicalensis*; HPLC-MS, high-performance liquid chromatography-mass spectrometry; RT, retention time.

We performed high-performance liquid chromatography-mass spectrometry (HPLC-MS) to identify baicalin, a constituent marker of SB, in the ethanol extract of SB (ESB). As displayed in Figure 1A, a baicalin peak was detected at a retention time of 12.977 min (Figure 1A and Table 1). The chromatogram of ESB also showed a peak at a retention time of 13.147 min similar to that of baicalin (Figure 1B and Table 1). The molecular weight of the indicated peak in the chromatogram of ESB and that of baicalin was the same as m/z 447.1 [M+H]^+^, suggesting that the indicated peak was baicalin (Table 1). To quantify the amount of baicalin, we further conducted HPLC-MS/MS analysis. According to the quantification data, ESB contained approximately 18% of baicalin (Table 1). The mass spectral and quantification data of baicalin is presented in Table 1.

### 2.2. Establishment of EGFR TKI-Resistant PC9 Cell Lines

**Figure 2:**
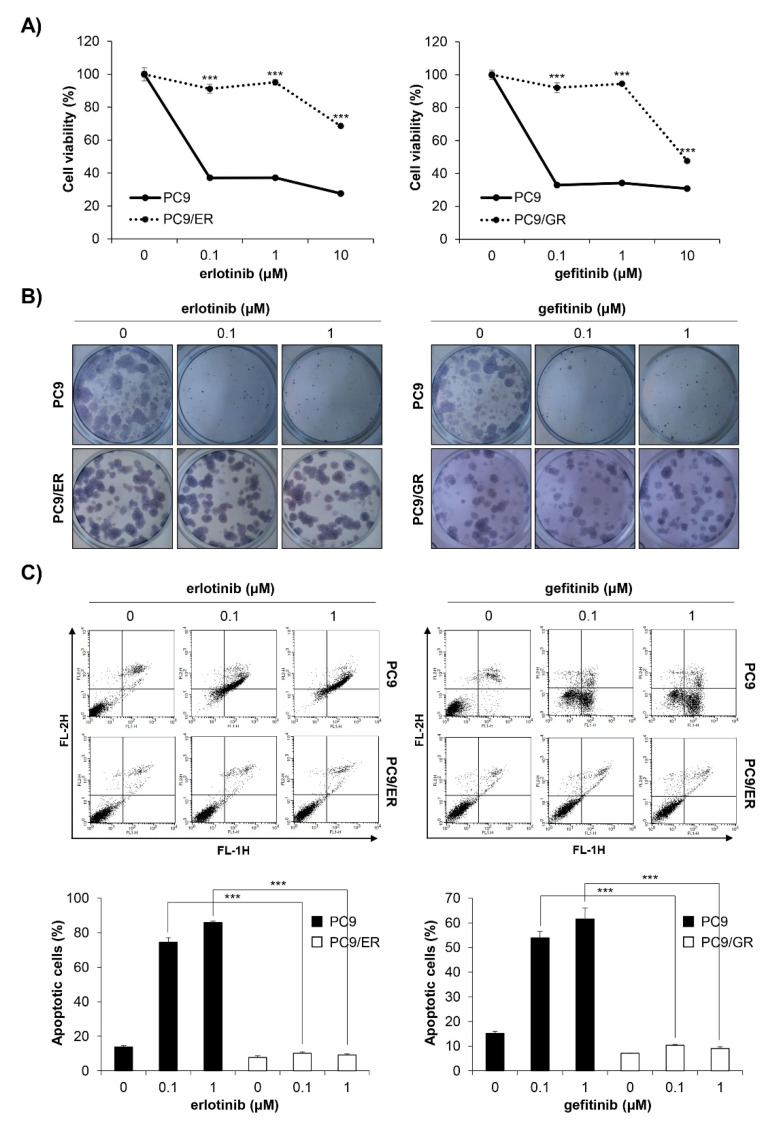
Establishment of PC9/ER and PC9/GR human NSCLC cell lines. (**A**) EGFR TKI-sensitive PC9, acquired erlotinib-resistant PC9/ER, and acquired gefitinib-resistant PC9/GR human NSCLC cell lines were treated with various concentrations of erlotinib (left panel) or gefitinib (right panel) for 72 h. The cell viability was measured by the MTT assay. (**B**) The cells were seeded as a single cell suspension onto 12-well plates and treated with different concentrations of erlotinib (left panel) or gefitinib (right panel) for 10 days. The colonies were photographed using a digital camera (upper panel) and the number of colonies was counted using ImageJ software (lower panel). (**C**) The cells were treated with the indicated concentrations of erlotinib (left panel) or gefitinib (right panel) for 72 h, and the annexin V- and/or PI_stained cells were analyzed by flow cytometry. Representative plot images are shown (upper panel). The percentage of annexin V-positive cells was measured by CellQuest software (lower panel). The data are expressed as the mean ± SD of three independent experiments. Significance was determined by the Student’s *t*-test (*** *p* < 0.001 vs. untreated controls). NSCLC, non-small-cell lung cancer; EGFR TKI, Epidermal growth factor receptor tyrosine kinase inhibitor; SD, standard deviation.

We established EGFR TKI-resistant cell lines by exposing EGFR TKI-sensitive PC9 human NSCLC cells to increasing concentrations of erlotinib or gefitinib. It took four months until stable resistant cell lines, referred to as PC9/ER (erlotinib-resistant PC9) and PC9/GR (gefitinib-resistant PC9), were established. To verify whether EGFR TKI resistance was well-generated, the MTT assay and anchorage-dependent colony formation assay were conducted. As shown in Figure 2A,B, the cell viability and colony-forming ability of the PC9/ER and PC9/GR cells were significantly higher than those of the PC9 cells following erlotinib or gefitinib treatment (Figure 2A,B). The annexin V-PI double staining assay results also showed that 72 h treatment with EGFR TKIs did not induce apoptosis in PC9/ER and PC9/GR cells, while it markedly increased the rate of apoptotic cells in PC9 cells (Figure 2C). Collectively, these results demonstrate that EGFR TKI-resistant cell lines were well-established. We also verified that H1299 *EGFR* wildtype cells and H1975 acquired EGFR TKI-resistant cells showed low sensitivity to erlotinib and gefitinib, which was proven by MTT assay (Supplementary Figure S1A,B).

### 2.3. Inhibition of Cell Growth by ESB in EGFR TKI-Resistant Cell Lines

**Figure 3:**
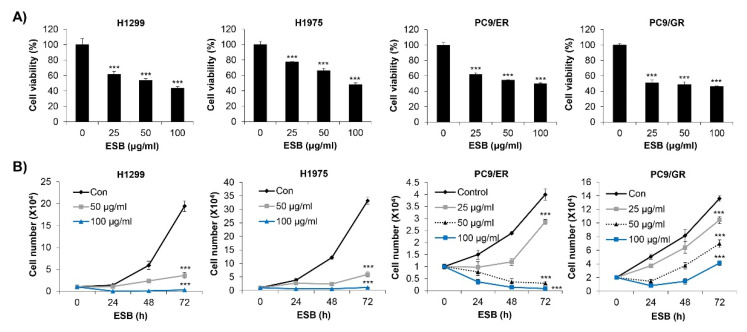
Effect of ESB on the cell growth of EGFR TKI-resistant human NSCLC cell lines. (**A**) H1299 (*EGFR* wildtype; EGFR TKI-resistant), H1975 (acquired TKI-resistant), PC9/ER (acquired erlotinib-resistant), and PC9/GR (acquired gefitinib-resistant) human NSCLC cell lines were treated with different concentrations of ESB for 72 h. Cell viability was measured by the MTT assay. (**B**) Cells were treated with the indicated concentrations of ESB for 24–72 h. The number of live cells was counted by the trypan blue exclusion assay. The data are expressed as the mean ± SD of three independent experiments. Significance was determined by the Student’s *t*-test (*** *p* < 0.001 vs. untreated controls). ESB, ethanol extract of the root of *Scutellaria baicalensis;* NSCLC, non-small-cell lung cancer; EGFR TKI, Epidermal growth factor receptor tyrosine kinase inhibitor; SD, standard deviation.

We first investigated the effects of ESB on the cell survival of EGFR TKI-resistant human NSCLC cell lines. We used four types of cell lines, including H1299 (*EGFR* wild-type; EGFR TKI-resistant), H1975 (*EGFR* L858R/T790M double-mutant; acquired EGFR TKI-resistant), PC9/ER (acquired erlotinib-resistant), and PC9/GR (acquired gefitinib-resistant) cell lines. The MTT assay results showed that ESB dose-dependently decreased the cell viability of the EGFR TKI-resistant cell lines (Figure 3A). We obtained the same results when cell viability was measured by the trypan blue exclusion assay. ESB suppressed the cell growth of EGFR TKI-resistant cell lines in time- and concentration-dependent manners (Figure 3B). Taken together, our results suggest that ESB inhibited the cell growth of EGFR TKI-resistant human NSCLC cells.

### 2.4. Inhibition of Colony Formation by ESB in EGFR TKI-Resistant Cell Lines

**Figure 4:**
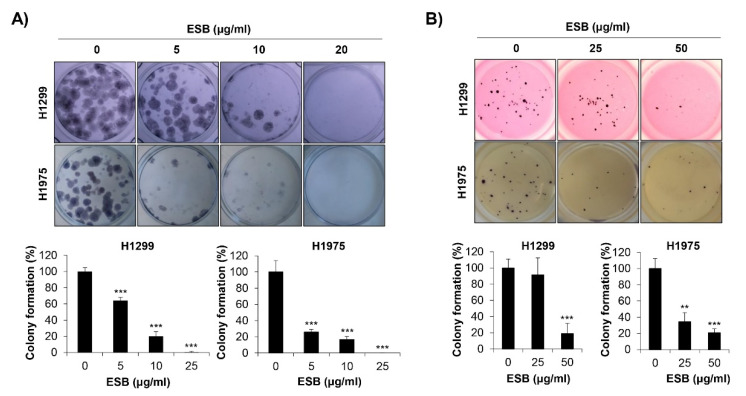
Effects of ESB on the colony formation of EGFR TKI-resistant human NSCLC cell lines. H1299 (*EGFR* wildtype; EGFR TKI-resistant) and H1975 (acquired TKI-resistant) human NSCLC cell lines were seeded as a single cell suspension in 12-well plates (**A**) or soft agar (**B**) and treated with the indicated concentrations of ESB for 10–15 days. The colonies were photographed by a digital camera (upper panel), and the number of colonies was counted using ImageJ software (lower panel). The data are expressed as the mean ± SD of three independent experiments. Significance was determined by the Student’s t-test (** *p* < 0.01, *** *p* < 0.001 vs. untreated controls). ESB, ethanol extract of the root of *Scutellaria baicalensis;* NSCLC, non-small-cell lung cancer; EGFR TKI, Epidermal growth factor receptor tyrosine kinase inhibitor; SD, standard deviation.

We next explored the effects of ESB on the colony-forming ability of EGFR TKI-resistant human NSCLC cell lines. As a pivotal step in tumorigenesis, cancer cells grow to form colonies. To mimic the tumorigenic environment, we performed the anchorage-dependent colony formation assay as well as the anchorage-independent soft agar assay. We observed that both the *EGFR* wildtype H1299 cells (EGFR TKI-resistant) and the acquired EGFR TKI-resistant H1975 cells formed colonies 10–15 days after seeding. As displayed in Figure 4A, ESB suppressed the anchorage-dependent colony formation in both cell lines (Figure 4A). In addition, the anchorage-independent colony formation was also obviously reduced by ESB treatment in a concentration-dependent manner (Figure 4B). Thus, our observations demonstrate that ESB suppressed the colony-forming ability of EGFR TKI-resistant human NSCLC cells.

### 2.5. Induction of Apoptosis by ESB in EGFR TKI-Resistant Cell Lines

**Figure 5:**
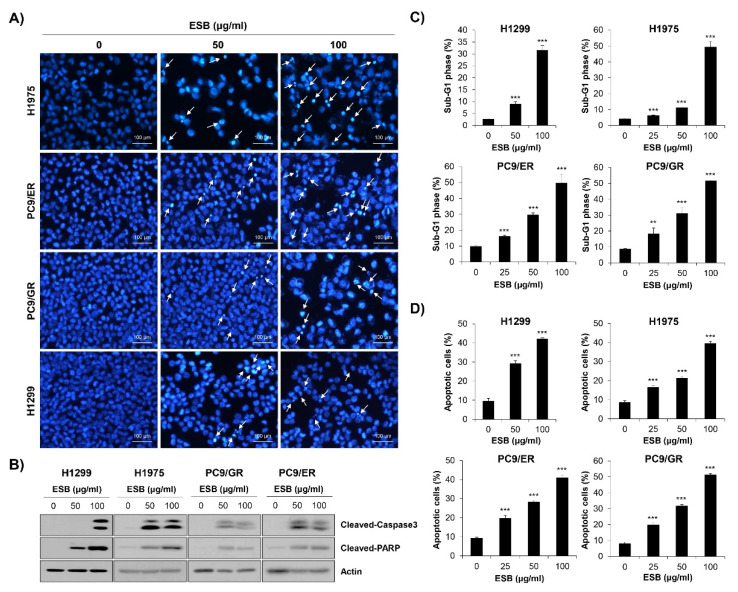
Induction of apoptosis by ESB in EGFR TKI-resistant human NSCLC cell lines. H1299 (*EGFR* wildtype; EGFR TKI-resistant), H1975 (acquired TKI-resistant), PC9/ER (acquired erlotinib-resistant), and PC9/GR (acquired gefitinib-resistant) human NSCLC cell lines were treated with different concentrations of ESB for 72 h. (**A**) The nuclear morphology visualized by DAPI staining was observed under a fluorescence microscope at ×200 magnification. The white arrows indicate apoptotic cells. The representative images of three independent experiments are shown. (**B**) The expression of cleaved caspase-3 and cleaved PAPR was detected by Western blot analysis. Actin was used as a loading control. (**C**,**D**) Cell cycle distribution (**C**) and annexin V-PI-double stained cells (**D**) were analyzed by flow cytometry. The sub-G1 DNA content (**C**) and percentage of annexin V+ cells (**D**) were measured by CellQuest software. The data are expressed as the mean ± SD of three independent experiments. Significance was determined by the Student’s *t*-test (** *p* < 0.01, *** *p* < 0.001 vs. untreated controls). ESB, ethanol extract of the root of *Scutellaria baicalensis*; NSCLC, non-small-cell lung cancer; EGFR TKI, Epidermal growth factor receptor tyrosine kinase inhibitor; STAT3, signal transducer and activator of transcription 3; SD, standard deviation.

To examine whether the inhibition of cell growth and colony formation by ESB was related to apoptosis induction, nuclear 4′,6-diamidino-2-phenylindole (DAPI) staining was performed. Chromatin condensation and nuclear fragmentation, typical markers of apoptosis, were dose-dependently increased by ESB treatment in EGFR TKI-resistant human NSCLC cell lines (Figure 5A). To validate the results of DAPI staining, Western blot analysis was conducted. The results showed that the cleavage of caspase-3 and PARP, apoptosis marker proteins, were upregulated by ESB in EGFR TKI-resistant cell lines (Figure 5B). We next monitored apoptosis by flow cytometry. The 72-h treatment of ESB markedly increased the sub-G1 population, generally considered to be apoptotic cells, in a concentration-dependent manner (Figure 5C and Supplementary Figure S2A). Cell cycle arrest at the G1/S- or G2/M-phase was not detected. In addition, the percentage of annexin V-positive apoptotic cells was also significantly increased by ESB in these cell lines (Figure 5D and Supplementary Figure S2B). Taken together, our results demonstrate that ESB triggered apoptosis in EGFR TKI-resistant human NSCLC cells.

Interestingly, the apoptosis induction by ESB was not consistently observed in EGFR TKI-sensitive PC9 cell line. As shown in Supplementary Figure S3A, the growth-inhibitory effect of ESB in PC9 cells was not as high as in the other EGFR TKI-resistant cell lines. Low concentration of ESB (25 μg/mL) did not show any cytotoxicity in PC9 cells, while the same concentration of ESB definitely suppressed the cell growth in EGFR TKI-resistant cell lines. In addition, the cell viability of PC9 cells after ESB treatment at 100 μg/mL was 57.28%, whereas that of EGFR TKI-resistant cell lines was ≤50% (Supplementary Figure S3A). Furthermore, the proportion of sub-G1 phase cells and annexin V+ cells was not increased by ESB in PC9 cells, indicating that ESB didn’t induce apoptosis in PC9 cell line (Supplementary Figure S3B,C). These results suggest that EGFR TKI-resistant cell lines show higher sensitivity to ESB than EGFR TKI-sensitive cell line.

### 2.6. Inactivation of STAT3 Mediates ESB-Induced Apoptosis in EGFR TKI-Resistant Cell Lines

**Figure 6:**
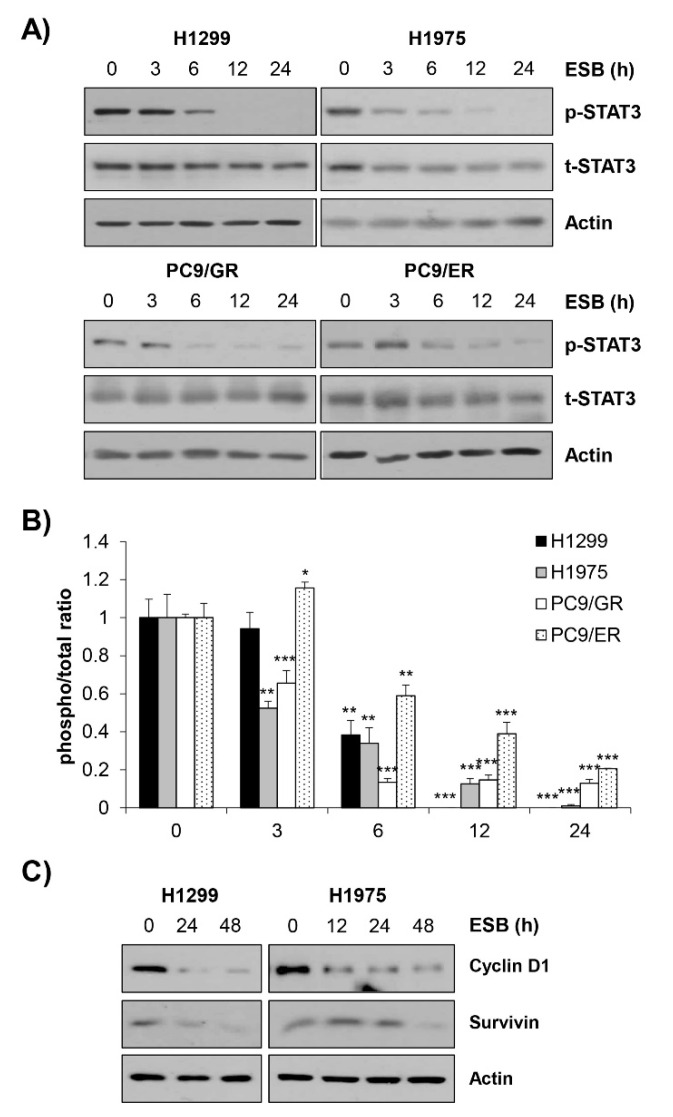
Suppression of STAT3 activity by ESB in EGFR TKI-resistant human NSCLC cell lines. H1299 (*EGFR* wildtype; EGFR TKI-resistant), H1975 (acquired TKI-resistant), PC9/ER (acquired erlotinib-resistant), and PC9/GR (acquired gefitinib-resistant) human NSCLC cell lines were treated with 100 µg/mL ESB for the indicated periods. (**A**) The levels of phosphorylated and total STAT3 were detected by Western blot analysis. Actin was used as a loading control. (**B**) The ratio of phosphorylated/total protein was calculated using ImageJ software after normalization to actin. The data are expressed as the mean ± SD of duplicate experiments. Significance was determined by the Student’s *t*-test (* *p* < 0.05, ** *p* < 0.01, *** *p* < 0.001 vs. untreated controls). (**C**) The protein expression of STAT3 target genes was detected by Western blot analysis. Actin was used as a loading control. ESB, ethanol extract of the root of *Scutellaria baicalensis;* NSCLC, non-small-cell lung cancer; EGFR TKI, Epidermal growth factor receptor tyrosine kinase inhibitor; STAT3, signal transducer and activator of transcription 3; SD, standard deviation.

We next explored the molecular mechanism underlying the ESB-induced apoptosis in EGFR TKI-resistant cell lines by focusing on the activity of STAT3. Given that STAT3 is frequently activated in NSCLC and controls major cancer properties, STAT3 has been considered a promising target for the treatment of NSCLC [25,26,27]. Interestingly, EGFR TKI-resistant tumors displayed increased STAT3 phosphorylation compared to EGFR TKI treatment-naïve or sensitive tumors [28,29,30]. Growing evidence suggests that STAT3 may be a key mediator of both primary and acquired resistance to EGFR TKIs [31]. Based on the previous studies, we hypothesized that ESB induced apoptosis in EGFR TKI-resistant cells by regulating STAT3 activity. Consistently with our hypothesis, ESB suppressed the phosphorylation of STAT3 in a time- and concentration-dependent manner in EGFR TKI-resistant human NSCLC cell lines (Figure 6A,B, Supplementary Figure S4). Consistently, the protein expression of cyclin D1 and survivin target genes transcriptionally regulated by STAT3, was diminished by ESB in a time-dependent manner, suggesting that ESB suppressed the transcriptional activity of STAT3 in EGFR TKI-resistant cell lines (Figure 6C).

**Figure 7:**
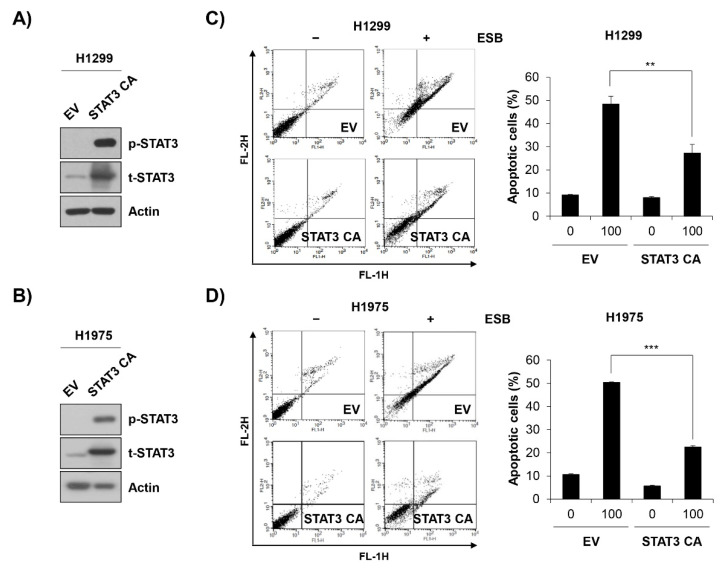
ESB-induced apoptosis in EGFR TKI-resistant human NSCLC cell lines was mediated by STAT3 activity suppression. H1299 cells and H1975 cells were transfected with constitutively active STAT3 (STAT3 CA) plasmid or empty vector. (**A**,**B**) At 48 h post-transfection, the levels of phosphorylated and total STAT3 in H1299 cells (**A**) and in H1975 (**B**) were detected by Western blot analysis. (**C**,**D**) At 48 h post-transfection, H1299 cells (**C**) and H1975 (**D**) were seeded again for annexin V-PI double staining assay and treated with ESB (100 µg/mL) for 72 h. The annexin V- and/or PI-stained cells were analyzed by flow cytometry. Representative plot images are shown (left panel). The percentage of annexin V-positive cells was measured by CellQuest software (right panel). The data are expressed as the mean ± SD of three independent experiments. Significance was determined by the Student’s *t*-test (** *p* < 0.01, *** *p* < 0.001 vs. untreated controls). ESB, ethanol extract of the root of *Scutellaria baicalensis;* NSCLC, non-small-cell lung cancer; EGFR TKI, Epidermal growth factor receptor tyrosine kinase inhibitor; STAT3, signal transducer and activator of transcription 3; SD, standard deviation.

To verify the role of STAT3 inactivation in ESB-induced apoptosis, EGFR TKI-resistant cell lines were transfected with constitutively active STAT3 (STAT3 CA) and treated with ESB. At 48 h post-transfection with STAT3 CA, H1299 and H1975 cells exhibited a strong expression of phospho-STAT3 compared to empty vector (EV)-transfected cells, suggesting that the STAT3 CA plasmid was successfully transfected (Figure 7A,B). Notably, the overexpression of STAT3 CA in H1299 and H1975 cells partially reversed the cytotoxicity of ESB. The percentage of apoptotic cells in EV-transfected cells was increased up to 48.38 ± 3.39% and 50.34 ± 0.25% in H1299 and H1975 cells, respectively, whereas that of STAT3 CA-transfected cells was only 27.23 ± 3.79% and 22.48 ± 0.63%, respectively, for each cell line (Figure 7C,D). In summary, our observations suggest that ESB triggered apoptosis in EGFR TKI-resistant human NSCLC cells by suppressing STAT3 activity.

Notably, the inhibitory effect of ESB on STAT3 activity was not observed in EGFR TKI-sensitive PC9 cell line (Supplementary Figure S3D). Given that ESB didn’t induce apoptosis in PC9 cells (Supplementary Figure S3B,C), these results support our conclusion that the anticancer effects of ESB is mediated by STAT3 inhibition.

## 3. Discussion

The current study investigated the molecular mechanism by which ESB induced apoptosis in EGFR TKI-resistant human NSLCL cell lines. The novelties of this research are as follows. First, we evaluated the anticancer effects of ESB in EGFR TKI-resistant human NSLCL cell lines for the first time. Although previous studies have reported that ESB triggered apoptosis and cell cycle arrest and inhibited the migration and invasion of human lung cancer cells [21,22,23,24], they did not focus on the activity of ESB in EGFR TKI-resistant cell lines. In this study, we used various cell lines with EGFR TKI-resistance, including H1299, H1975, PC9/ER, and PC9/GR cells, to access whether ESB could be used for the treatment of NSCLC with EGFR TKI resistance. Our results demonstrated that ESB reduced cell growth and colony formation in EGFR TKI-resistant cells by inducing apoptosis.

Second, to the best of our knowledge, this was the first study to illustrate that ESB regulated STAT3 activity. No study has reported the role of STAT3 in the pharmacological activity of the crude extract of SB, including anticancer effects. The reason we postulated STAT3 as a potential target of ESB was based on the following points: (i) STAT3 frequently overactivated in NSCLC contributed to cancer progression by regulating major cancer hallmarks, such as cell proliferation, angiogenesis, metastasis, the evasion of immune surveillance, and chemoresistance [25,26,27], (ii) the level of STAT3 phosphorylation was higher in the EGFR TKI-resistant tumors than in the EGFR TKI-naïve or -sensitive tumors [28,29,30], and (iii) previous studies postulated STAT3 as a key mediator of both primary and acquired resistance to EGFR TKIs [31]. Our results showed that STAT3 was dephosphorylated by ESB in EGFR TKI-resistant human NSCLC cells. In addition, the overexpression of constitutively active STAT3 reversed ESB-induced apoptosis. These results collectively demonstrate that the inactivation of STAT3 was involved in the anticancer activity of ESB.

It has been suggested that a variety of pathways are involved in the anticancer effects of SB and its constituents [19]. For example, NF-κB was inactivated by SB extract and its constituents, including baicalein or wogonin [32,33,34]. According to our preliminary data, p65 NF-κB phosphorylation was not decreased, and even slightly increased by ESB in EGFR TKI-resistant cells, suggesting that NF-κB was not involved in ESB-induced apoptosis. The dephosphorylation of AKT and mTOR protein is also reported to mediate the anticancer effects of SB and its constituents [35,36,37,38]. However, our preliminary data showed that while phosphorylated AKT was slightly downregulated until 12 h posttreatment with ESB, it recovered to control levels at 24 h, dismissing the possibility that AKT inactivation mediated the anticancer effects of ESB. ERK has been reported as another target of SB. ERK was inactivated by baicalein, wogonin, and oroxylin A to inhibit cell growth, migration, and the invasion of cancer cells [39,40,41].

Consistently, our preliminary data also showed that ERK phosphorylation was commonly decreased by ESB in EGFR TKI-resistant cell lines, suggesting that ERK can be another candidate involved in ESB-induced apoptosis. This might also explain why the overexpression of constitutively active STAT3 in EGFR TKI-resistant cells did not completely reverse ESB-induced apoptosis. In that case, other upstream ERK kinases, except for STAT3, would have regulated the activity of ERK in a compensatory mechanism. However, it can be still possible that the activity of STAT3 was partially suppressed by ESB even after the transfection of constitutively active STAT3, resulting in the incomplete reversal of ESB-induced apoptosis.

In a future study, the specific ESB compound that causes apoptosis in EGFR TKI-resistant cell lines should be determined. Previous studies have reported that several active compounds of SB suppressed STAT3 activity. For example, baicalin inhibited amyloid β-induced microglial cell activation and promoted neuronal differentiation of neural stem cells by inhibiting STAT3 phosphorylation [42,43]. Another constituent of SB, baicalein attenuated metastatic potential of breast cancer cells by regulating STAT3 activity [44]. Oroxylin A also decreased STAT3 activity to suppress colitis-stimulated carcinogenesis [45]. Therefore, we propose these compounds as putative ingredients for exerting anticancer effects in the EGFR TKI-resistant cell lines. However, whether other compounds of SB can modulate the STAT3 signaling pathway should be extensively investigated. Whether ESB attenuates cell growth and triggers apoptosis in other cancer cell types with EGFR TKI resistance, such as pancreatic and breast cancer cells, is also an important issue in predicting the possibility of applying ESB to various cancer types [40]. Interestingly, our results showed that ESB did not induce apoptosis, nor did ESB suppress STAT3 phosphorylation in the EGFR TKI-sensitive PC9 cell line. Whether ESB shows selectivity for EGFR TKI-resistant cells and the precise mechanism should be further investigated in the future study.

Taken together, our results showed that ESB reduced cell growth and induced apoptosis in EGFR TKI-resistant human NSCLC cell lines by suppressing STAT3 activity. We provide basic information about the potential anticancer effects of ESB in EGFR TKI-resistant NSCLC with highly activated STAT3.

## 4. Materials and Methods

### 4.1. Preparation of ESB

The dried root of SB was purchased from Bonchomaru (Seoul, Korea). SB (50 g) was pulverized into powder and extracted with 800 mL of 80% ethanol at 40 °C with shaking (150 rpm). After 72 h, the extract was collected, and the SB was extracted again with 300 mL of 80% ethanol under the same conditions. After 24 h, the extract was combined with the previous one, concentrated by a vacuum rotary evaporator under reduced pressure, and lyophilized. The yield was 42.28%. The ESB used in the experiments was prepared by dissolving the powder in dimethyl sulfoxide (DMSO; Amresco, Solon, OH, USA) at 200 mg/mL as a stock solution.

### 4.2. HPLC-MS/MS Analysis

HPLC analysis was conducted on a Dionex UltiMate 3000 UHPLC system (Thermo Fisher Scientific, San Jose, CA, USA) using Thermo Chromeleon 7 software (Thermo Fisher Scientific, San Jose, CA, USA). Baicalin (ChemFaces, Wuhan, China) was dissolved in 10% DMSO in methanol. The separation was performed on a YMC Triart C18 column (150 × 2.0 mm, 3 μm) using 1% acetic acid in distilled water (DW) and 1% acetic acid in 70% acetonitrile as solvent A and solvent B, respectively. The mobile conditions were as follows: 75% solvent A and 25% solvent B for 10 min, 68% solvent A for 10 min, 55% solvent A for 10 min, 55% solvent A for 4 min, 52% solvent A for 11 min, 75% solvent A for 5 min, and holding for 5 min. The flow rate was 0.2 mL/min and the column temperature was maintained at 40 °C. The detection wavelength was 275 nm. MS analysis was performed on a Compact mass spectrometer LC-MS system (Advion, Ithaca, NY, USA). The mass spectra were recorded over m/z 100–1200 with a scan speed of 1100 (scan time).

To quantify the amount of baicalin, HPLC-MS/MS analysis was performed. HPLC analysis was carried out using a 1200 series LC system equipped with a G1322A degasser, G1312A pump, a G1367D autosampler and a G1316A oven (Agilent Technologies, Palo Alto, CA, USA). Chromatographic separation was performed on a InfinityLab Poroshell 120 EC- C18 column (2.1 × 100 mm, 2.7 µm, Agilent Technologies, Folsom, CA, USA). The binary solvent system consisted of 0.1% Formic acid, 5 mM Ammonium Formate in DW (A) and 0.1% Formic acid, 5 mM Ammonium Formate in Methanol (B). The mobile conditions were as follows: 80% solvent A and 20% solvent B for 1 min, 5% solvent A for 1.1 min, and holding 5.4 min, 80% solvent A for 6.6 min, and holing 3.4 min. The flow rate was 0.3 mL/min with a column temperature of 40 °C and an injection volume of 2 μL in each experiment. Chromatographic data were collected and manipulated using Agilent MassHunter, B.06.00 (Agilent Technologies, Folsom, CA, USA). MS/MS experiments were conducted using a 6410 Triple Quad LC-MS system (Agilent Technologies) with a ESI source. The mass spectrometer was operated in the positive mode with selected Multiple Reaction Monitoring (MRM). Agilent MassHunter (version B.06.00.) were used for instrumental control and data acquisition. Nitrogen at a pressure of 30 psi was used as a nebulizer. The gas temperature was 350 °C, and the capillary voltage was 4 kV. The precursor ion of the baicalin were 447.1 m/z. The product ion of the baicalin are 271.1 m/z and 123 m/z, and collision energy values are 30 V and 21 V, respectively. The amount of baicalin in ESB was quantified by three replicates determinations.

### 4.3. Cell Culture

H1299, H1975, and PC9 human NSCLC cell lines were kind gifts from Professor Ho-Young Lee (Seoul National University, Seoul, Korea). RPMI-1640 (WelGENE, Daegu, Korea) with 10% fetal bovine serum (FBS; WelGENE, Daegu, Korea) and 1% antibiotics (WelGENE, Daegu, Korea) was used as the culture medium. The cells were sub-cultured every three days and maintained at 37 °C under 5% CO_2_. Erlotinib-resistant PC9 (PC9/ER) and gefitinib-resistant PC9 cell lines (PC9/GR) were established by exposing PC9 cells to increasing concentrations of erlotinib (LC Laboratories, Woburn, MA, USA) or gefitinib (Cayman Chemical, Ann Arbor, MI, USA). The PC9 cells were treated with 0.1 μM of erlotinib or gefitinib as a starting concentration. The culture medium was changed to fresh medium every three days until the cells reached confluence. The cells were then incubated with gradually increasing concentrations of EGFR TKIs for four months. The final concentrations of the drugs were 50 μM for erlotinib and 25 μM for gefitinib. The PC9/ER and PC9/GR cell lines were cultured in medium containing erlotinib (25 μM) or gefitinib (12.5 μM), respectively.

### 4.4. MTT Assay

Cells (3 × 10^3^) were plated into 96-well plates and stabilized overnight. The culture medium was replaced with fresh medium (120 μL) containing the indicated drugs. After 72 h of incubation, 120 μL of MTT [3-(4,5-dimethylthiazol-2-yl)-2,5-diphenyltetrazolium bromide; Duchefa, Haarlem, The Netherlands] solution (4 mg/mL) was added to each well. After 2 h of incubation at 37 °C, the culture media was suctioned from the plates. Then, 100 μL of DMSO was added to each well as an MTT solvent. The plates were shaken on an orbital shaker for 10 min to fully solubilize the formazan crystals. The absorbance of each well at 540 nm was measured using a microplate reader (SpectraMax M3; Molecular Devices, San Jose, CA, USA).

### 4.5. Trypan Blue Exclusion Assay

Cells (2 × 10^4^) were plated in 12-well plates and stabilized overnight. The cells were treated with various concentrations of ESB and collected 24–72 h posttreatment with ESB. The harvested cells were washed and resuspended in 1 mL of phosphate-buffered saline (PBS, WelGENE, Daegu, Korea). Then, 0.1 mL of 0.4% trypan blue solution (WelGENE, Daegu, Korea) was added to 0.1 mL of cell suspension. The number of live cells was counted using a hemocytometer under a microscope (Leica, Wetzlar, Germany) by excluding the blue-stained cells.

### 4.6. Colony Formation Assay

For the anchorage-dependent colony formation assay, 2 × 10^2^ cells were plated in 12-well plates. After stabilizing overnight, the cells were treated with the indicated drugs. The cells were incubated at 37 °C until colonies were fully formed and grown, and the culture medium was replaced with fresh medium every three days. Ten days posttreatment, the colonies were fixed with 100% methanol for 5 min, stained with hematoxylin (Sigma-Aldrich, St. Louis, MO, USA) for 30 min, and washed with distilled water before obtaining images. For the anchorage-independent soft agar assay, 4% SeaPlaque agarose (Lonza, Rockland, ME, USA) was diluted with warm culture media at a ratio of 1:3 to make 1% bottom agar and overlaid on each well of 24-well plates. After the bottom agar was solidified, 0.4% agar (top agar) containing 1 × 10^3^ cells was added onto the bottom agar and left to solidify at room temperature. Then, 0.5 mL of warm culture media containing the indicated concentrations of ESB was added onto the top agar. The cells were incubated at 37 °C until colonies were fully formed and grown, and the culture media was replaced with fresh medium every three days. After 15 days of incubation, MTT solution (4 mg/mL) was added to the culture media at 0.5 mg/mL. The colonies were stained with MTT solution for 2 h at 37 °C. Images of the colonies were taken with a digital camera (Canon, Tokyo, Japan) and the number of colonies was measured using ImageJ software (version 1.52a).

### 4.7. DAPI Staining

Cells (1 × 10^5^) were plated in 6-well plates, stabilized overnight, and challenged with different concentrations of ESB. The cells were harvested and fixed with 3.7% paraformaldehyde (Sigma-Aldrich) for 30 min at 4 °C. After washing with cold PBS, the cells were attached to slide glasses using a Cytospin (Shandon Inc., Pittsburgh, PA, USA). Then, the cells were stained with 2.5 μg/mL DAPI solution for 20 min at room temperature in the dark for nuclear staining, washed with PBS, and mounted with mounting solution (Biomeda, Foster City, CA, USA). The nuclei were observed under fluorescence microscopy at ×200 magnification (Carl Zeiss, AG, Germany).

### 4.8. Flow Cytometry

Cells (1 × 10^5^) were seeded in 6-well plates, stabilized overnight, and treated with the indicated drugs. The cells were collected and subjected to cell cycle analysis. The cells were treated with cold 80% ethanol for 1 h at 4 °C for fixation and stained with 50 µg/mL propidium iodide (PI) solution (Sigma-Aldrich, St. Louis, MO, USA) containing 30 µg/mL RNase A (Sigma-Aldrich, St. Louis, MO, USA) for 30 min in the dark. After centrifugation, the supernatant was discarded, and the cells were resuspended in 500 µL of PBS. The percentage of cells in each phase of the cell cycle was measured by flow cytometry (FACSCaliber, Becton Dickinson and Company, San Jose, CA, USA). The cells in the sub-G1 fraction were considered apoptotic cells. For the annexin V-PI double-staining assay, the cells were stained with both annexin V-FITC and PI using the Annexin V-FITC Apoptosis Detection Kit I (BD Biosciences Pharmingen, San Diego, CA, USA) as described by the manufacturer. Then, the annexin- and/or PI-stained cells were measured by flow cytometry. The annexin V+ cells were considered apoptotic cells.

### 4.9. Western Blots

Cells (2 × 10^5^) were seeded in 60-mm dishes and treated with the indicated drugs. The cells were collected and lysed for 1 h on ice ibitor cocktail (Thermo Fisher Scientific, San Jose, CA, USA) and phosphatase inwith cold RIPA buffer (Thermo Fisher Scientific, San Jose, CA, USA) with added protease inhhibitors (1mM Na_3_VO_4_ and 100 mM NaF). After centrifugation at 16,000× *g* at 4 °C for 30 min, the protein concentration of the supernatant was measured using the bicinchoninic acid (BCA) protein assay kit (Pierce Biotechnology, Rockford, IL, USA). Protein (20 µg) from each sample was subjected to sodium dodecyl sulfate (SDS)-polyacrylamide gel electrophoresis and transferred to polyvinylidene fluoride (PVDF) membranes. After blocking with 3% bovine serum albumin (BSA, GenDEPOT, Barker, TX, USA) for 30 min at room temperature, the membranes were washed with TBST [Tris-buffered saline (TBS) supplemented with 0.1% Tween-20] for 10 min and probed with the specific primary antibodies at 1:1000 dilutions overnight at 4 °C. The membrane was then washed with TBST for 1 h and treated with the appropriate secondary antibody solution (1:10,000 dilution in 3% skim milk) for 1 h at room temperature. Protein expression was detected by the D-Plus ECL Femto System (Donginbio, Seoul, Korea). The primary antibodies against cyclin D1 and actin were purchased from Santa Cruz Biotechnology (Santa Cruz, CA, USA) and the other primary antibodies were all purchased from Cell Signaling Technology (Beverly, MA, USA). The goat anti-mouse secondary antibody and the goat anti-rabbit secondary antibody were purchased from Bethyl Laboratories (Montgomery, TX, USA) and Enzo Life Sciences (Farmingdale, NY, USA), respectively.

### 4.10. Transfection

Cells (3 × 10^5^) were plated in 6-well plates and stabilized overnight. The cells were transfected with 1 μg of constitutively active STAT3 plasmid (pExpress-STAT3Y705D) using 3 μg of Lipofectamine 2000 (Invitrogen, Carlsbad, CA, USA) as described in the manufacturer’s protocol. The constitutively active STAT3 plasmid was a gift from Professor Ho-Young Lee (Seoul National University, Seoul, Korea). After 48 h, the cells were harvested for Western blot analysis or seeded again for flow cytometry.

### 4.11. Statistical Analyses

Each result is expressed as the mean ± SD of data obtained from triplicate experiments. The statistical analysis was performed by a paired Student’s *t*-test. Differences at *p* < 0.05 were considered statistically significant.

## Figures and Tables

**Table 1:** The spectral data and quantitative value of baicalin and ESB peak.

Name	RT ^1^	MS ^2^ [M+H]^+ ^ (m/z)	MS/MS (m/z)	Quantity of Baicalin in ESB ± SD ^3^ (g/kg)
**Baicalin**	12.977	447.1 447.1	271.1 123.0	187.76 ± 1.06
**ESB peak**	13.147		271.1 123.0	

^1^ RT: retention time, ^2^ MS: mass spectrometry, ^3^ SD: standard deviation.

## Data Availability

The data presented in this study are available on request from the corresponding author.

## Supplementary Materials

The following are available online at https://www.mdpi.com/article/10.3390/ijms22105181/s1, Figure S1: Effects of EGFR TKIs on the cell viability of H1299 and H1975 cells, Figure S2: Representative images of flow cytometry, Figure S3: Effects of ESB on apoptosis induction and STAT3 activity in EGFR TKI-sensitive PC9 cell line, Figure S4: Concentration-dependent suppression of STAT3 by ESB in EGFR TKI-resistant human NSCLC cell lines.
