# Supplementary material for: The Root Extract of Scutellaria baicalensis Induces Apoptosis in EGFR TKI-Resistant Human Lung Cancer Cells by Inactivation of STAT3
Source: Int J Mol Sci. 2021 May 13;22(10):5181. doi: 10.3390/ijms22105181 (PMC8153615; doi:10.3390/ijms22105181)
Supplement: Supplementary file 1 [file ijms-22-05181-s001.zip › ijms-1169979-supplementary.pdf]

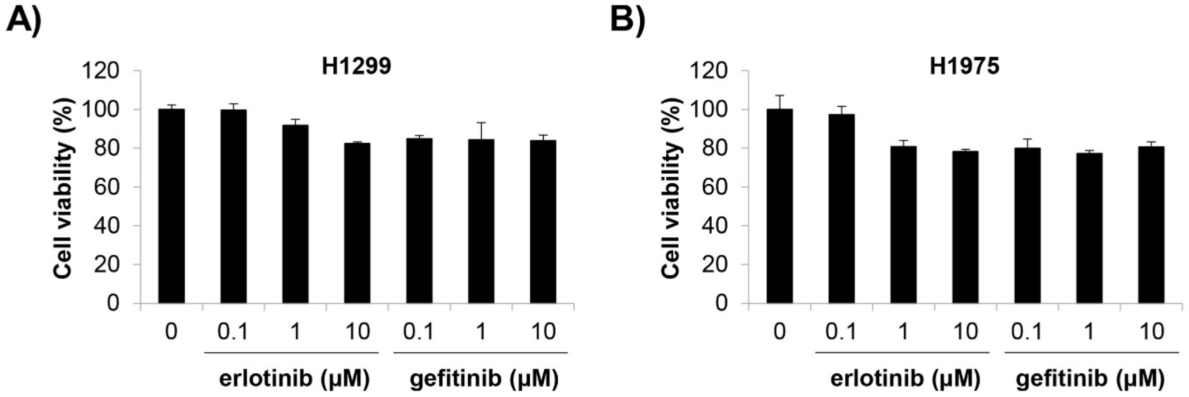

Figure S1. Effects of EGFR TKIs on the cell viability of H1299 and H1975 cells. H1299 primary EGFR TKI-resistant cells (A) and H1975 acquired EGFR TKI-resistant cells (B) were treated with various concentrations of erlotinib or gefitinib for 72 h. The cell viability was measured by the MTT assay. The data are expressed as the mean ± SD of three independent experiments. EGFR TKI, Epidermal growth factor receptor tyrosine kinase inhibitor; SD, standard deviation.

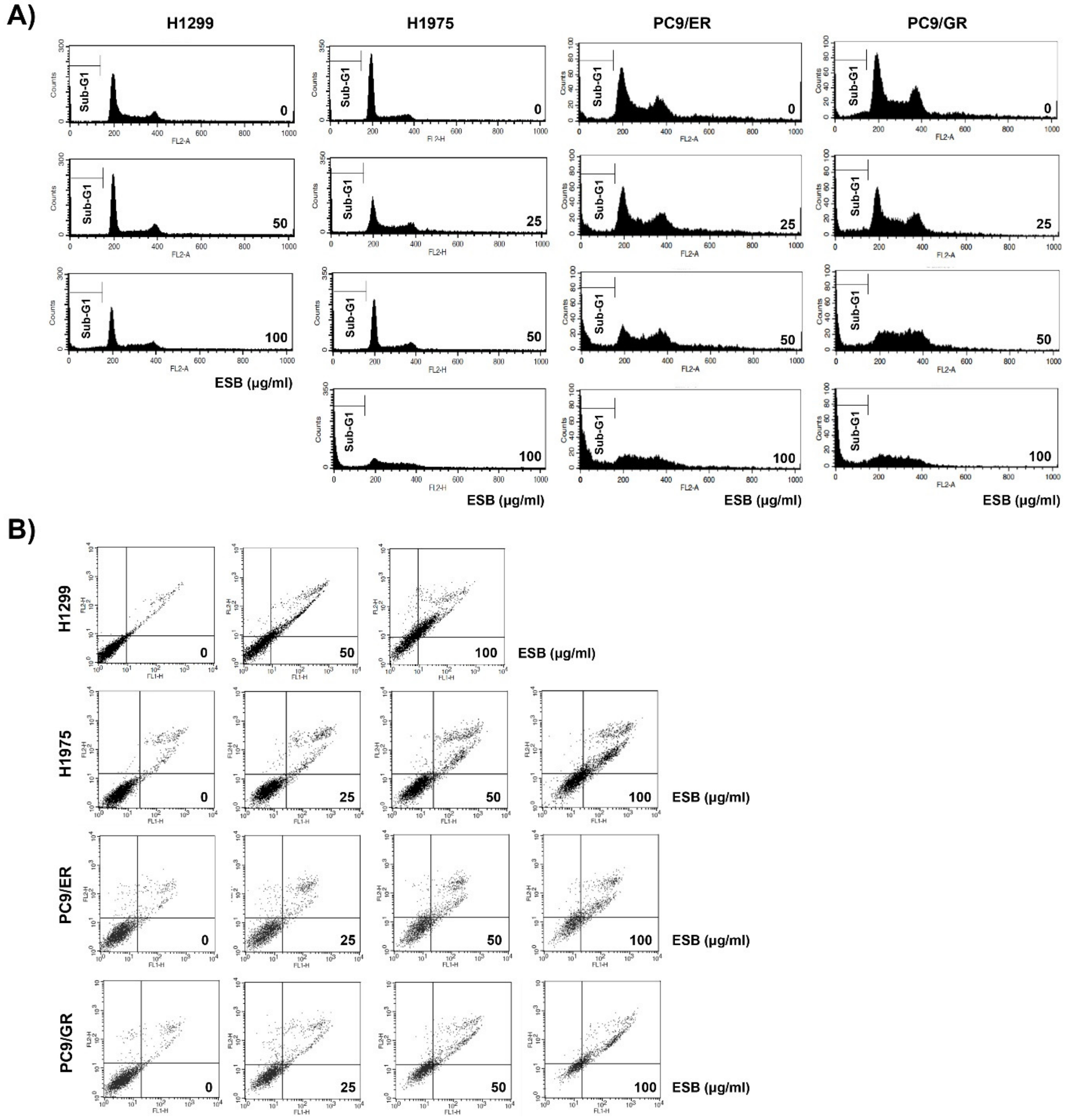

Figure S2. Representative images of flow cytometry. H1299 (EGFR wildtype; EGFR TKI-resistant), H1975 (acquired TKI-resistant), PC9/ER (acquired erlotinib-resistant), and PC9/GR (acquired gefitinib-resistant) human NSCLC cell lines were treated with different concentrations of ESB for 72 h. Representative histograms of cell cycle distribution (A) and dot plots of annexin V-PI double staining assay (B) were shown. ESB, ethanol extract of the root of *Scutellaria baicalensis*; NSCLC, non-small-cell lung cancer; EGFR, Epidermal growth factor receptor; TKI, tyrosine kinase inhibitor.

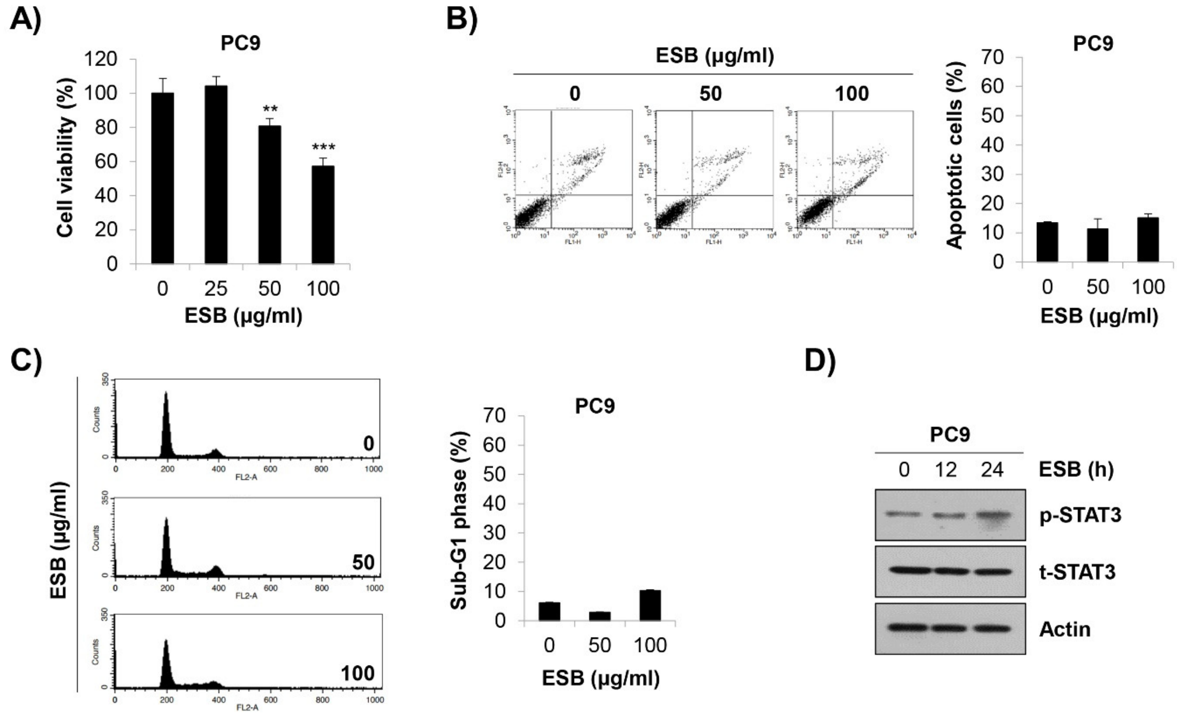

Figure S3. Effects of ESB on apoptosis induction and STAT3 activity in EGFR TKI-sensitive PC9 cell line. (A-C) EGFR TKI-sensitive PC9 cells were treated with different concentrations of ESB for 72 h. (A) The cell viability was measured by the MTT assay. (B) Apoptosis was detected by annexin V-PI double staining assay. The annexin V(+) cells were considered apoptotic cells. (C) Apoptosis was assessed by sub-G1 analysis. (D) PC9 cells were treated with ESB (100 µg/ml) for the indicated periods. The levels of phosphorylated and total STAT3 were detected by Western blot analysis. Actin was used as a loading control. The data are expressed as the mean ± SD of three independent experiments. Significance was determined by the Student's t-test (** $P < 0.01$, *** $P < 0.001$ vs. untreated controls). ESB, ethanol extract of the root of *Scutellaria baicalensis*; NSCLC, non-small-cell lung cancer; EGFR TKI, Epidermal growth factor receptor tyrosine kinase inhibitor; STAT3, signal transducer and activator of transcription 3; SD, standard deviation.

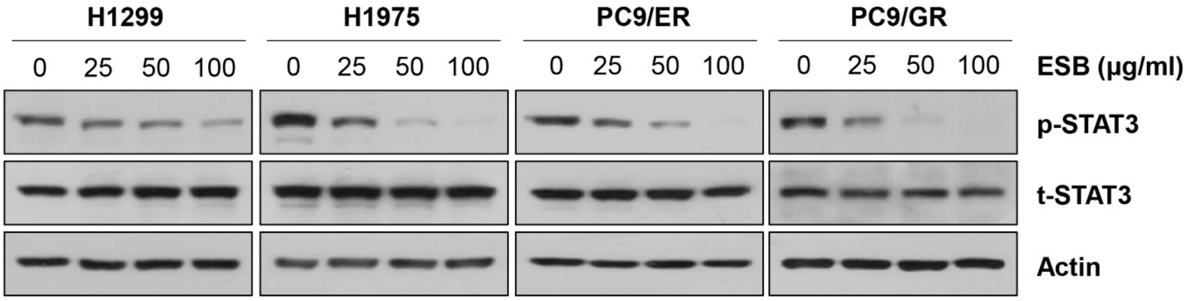

Figure S4. Concentration-dependent suppression of STAT3 by ESB in EGFR TKI-resistant human NSCLC cell lines. H1299 (EGFR wildtype; EGFR TKI-resistant), H1975 (acquired TKI-resistant), PC9/ER (acquired erlotinib-resistant), and PC9/GR (acquired gefitinib-resistant) human NSCLC cell lines were treated with different concentrations of ESB for 24 h. The levels of phosphorylated and total STAT3 were detected by Western blot analysis. Actin was used as a loading control. ESB, ethanol extract of the root of *Scutellaria baicalensis*; NSCLC, non-small-cell lung cancer; EGFR TKI, Epidermal growth factor receptor tyrosine kinase inhibitor; STAT3, signal transducer and activator of transcription 3.
